# Patient-centred workload clustering in adults under 65 with long-term conditions from longitudinal primary care data

**DOI:** 10.1136/bmjph-2026-005763

**Published:** 2026-10-30

**Authors:** Marc Delord, Emilia Holland, Mozhdeh Shiranirad, Alex Dregan, Simon D S Fraser

**Affiliations:** 1 Department of Population Health Sciences, King’s College London, London, UK; 2 School of Primary Care, Population Sciences and Medical Education, Faculty of Medicine, University of Southampton, Southampton, UK; 3 School of Mathematical Sciences, University of Southampton, Southampton, UK; 4 Department of Psychological Medicine, Institute of Psychiatry, Psychology and Neuroscience, King’s College London, London, UK; 5 Applied Research Collaboration Wessex, NIHR, Southampton, UK; 6 University Hospital Southampton NHS Foundation Trust, NHS, Southampton, UK

**Keywords:** Public Health, Epidemiology, Community Participation, Epidemiologic Measurements

## Abstract

**Introduction:**

Although patient workload is increasingly recognised as a relevant dimension of the patient experience, it is not satisfactorily captured in current electronic health records. We set out to describe the distribution of patient workload patterns across long-term conditions and demographic groups using electronic health records annotated to reflect patient-centred workload derived from a qualitative evidence synthesis of patient experience.

**Methods:**

We used a predefined compendium of 63 workload indicators, derived from a previously published qualitative evidence synthesis of patient experience and mapped to electronic health record codes, classified into eight themes. We computed counts of these indicators in 765 930 patients born between 1956 and 1965 with two or more recorded long-term conditions, randomly sampled from the Clinical Practice Research Datalink. We then used an unsupervised learning approach to construct a typology of patients based on these counts.

**Results:**

Median age at first record was 31 years with 28 years of follow-up. The cohort was 49.4% (n=378 154) male and predominantly white (68.1%, n=521 859). Patients had a median of 15 workload indicators and 4 long-term conditions. Nine clusters were identified, with marked contrasts in workload intensity, long-term conditions, demographic profiles and size. While large clusters were less burdensome, smaller clusters showed more complex profiles, characterised by cardiometabolic or mental health long-term conditions, higher female-to-male ratios and greater ethnic diversity. The most burdensome cluster, characterised by a broad spectrum of rarer long-term conditions and dominated by indicators associated with investigation and treatment monitoring, appeared comparatively less complex.

**Conclusions:**

This unsupervised approach to patient workload revealed patient clusters with marked disparities in burden, long-term conditions, demographic characteristics and complexity, supporting future research directions aimed at better understanding and supporting patients with elevated workload demands.

**WHAT IS ALREADY KNOWN ON THIS TOPIC**

- Treatment burden represents a complementary dimension of the patient experience that is not captured by disease-oriented representations in electronic health records.

**WHAT THIS STUDY ADDS**

- Using routine electronic health records annotated to represent patient workload based on a predefined framework, this study identifies distinct workload patterns that do not reduce to disease-oriented representations, with the highest workload not aligned with the most clinically complex patients.

**HOW THIS STUDY MIGHT AFFECT RESEARCH, PRACTICE OR POLICY**

- Workload-based stratification identifies patients with high care burden who are missed by disease counts, suggesting the need to develop and evaluate strategies to better address this burden.

## Introduction

 Health systems globally are facing a growing challenge from the increasing prevalence of long-term conditions and multimorbidity, driven by population ageing and earlier onset of chronic disease.^1^ This shift is placing sustained pressure on healthcare delivery, coordination and workforce capacity, and has raised legitimate concerns about the sustainability of existing health system models.^2^ In response, policy and clinical analyses have highlighted the limitations of disease-focused approaches and called for more integrated, patient-centred models of care for people living with multiple long-term conditions.^3–5^

In this context, increasing attention has been brought to patient workload and patient complexity in the experience of living with multiple long-term conditions, including treatment demands, disease burden and broader psychosocial factors.^6,7^ Annotating electronic health records to represent patient workload enables the capture of aspects of care not fully reflected in condition-based descriptions of multimorbidity, providing a complementary perspective on patient experience and extending the characterisation of patient complexity beyond disease-based descriptions and their consequences for health outcomes.^8^

However, while patient work—understood as treatment and disease burden—has entered conceptual models of patient complexity^6,9^ and has been associated with poorer quality of life, adverse outcomes and inefficiencies in care,^10,11^ patient complexity itself remains incompletely characterised and only partially operationalised in empirical research. In practice, it is most commonly approximated through counts of long-term conditions, which capture clinical burden, but overlook treatment demands, care coordination and psychosocial or financial workload.^8^ The need therefore arises to further clarify this concept and to confront theoretical frameworks of patient work and complexity with routinely collected primary care data.^12,13^ To better characterise and quantify patient workload from a patient perspective, a qualitative evidence synthesis, grounded in patient-reported experiences, identified eight patient-centred themes of work.^7^ These were subsequently translated into structured workload indicators mapped to routine electronic health records.^13^

This work adopts a patient perspective that is complementary to healthcare system-oriented approaches, in which multimorbidity has primarily been analysed through its implications for healthcare utilisation, often linking counts of long-term conditions to levels of service use.^14–16^ It also contrasts with disease-oriented clustering studies that group individuals by long-term condition profiles, and whose alignment with patient workload remains unclear.^4,17^ By mapping workload indicators and themes to sets of routinely collected primary care electronic health record codes reflecting these themes, we propose in this study an operational, patient-centred approach to characterising long-term conditions in terms of patient workload. This is particularly relevant in midlife, when the accumulation of conditions and care demands can substantially increase workload, with downstream consequences for health trajectories and capacity to manage care, as well as implications for patient-centred service design, healthcare planning and medical education.^2,12,18^ Despite this recent attention, common workload patterns and their distribution across long-term conditions and demographic groups remain poorly characterised, limiting understanding of patients’ needs and complexity.

In response to these challenges, and building on prior conceptual and methodological work on patient workload,^7,13^ this study identifies clusters of patients derived from counts of workload indicators using unsupervised learning applied to routinely collected primary care electronic health records, and describes these clusters in terms of demographic characteristics, long-term conditions and workload indicators and themes. By focusing on patient workload, this approach moves beyond simple counts of long-term conditions and disease-based descriptions of multimorbidity to better characterise the complexity of living with multiple long-term conditions, in line with identified research priorities.^5^

## Methods

### Study population

We used data from the Clinical Practice Research Datalink (CPRD) Aurum, December 2024 release—a large longitudinal database of routinely collected primary care electronic health records in England and Northern Ireland.^19^ Covering approximately 17 million actively registered patients and around one-fifth of English general practices, CPRD Aurum is broadly representative of the UK primary care population by age, sex, socioeconomic status and ethnicity.^20,21^ The database includes longitudinal data on diagnoses, prescriptions, laboratory results, referrals and lifestyle factors.^22^

We analysed a random 33% sample of individuals born between 1 January 1956 and 31 December 1965. Individuals were eligible for inclusion if they had at least two recorded long-term conditions during the observation period. Follow-up extended from the first recorded primary care record to the last available record or death and was restricted to records meeting CPRD research-quality standards.^23^ Workload indicators were accumulated across the entire observation period and were not restricted to the period following the second recorded long-term condition. Sex was recorded as male or female and included in all analyses.

### Definition and counts of workload indicators and long-term conditions

The qualitative evidence synthesis identified eight patient-centred themes of work—Learning and Adapting, Accumulation and Complexity, Investigation and Monitoring, Health Service and Administration, Symptom work, Emotional work, Medication work and Financial work.^7^ These themes were subsequently translated into 63 indicators, mapped to 10 263 SNOMED CT clinical codes.^13^ For each patient, workload indicator occurrences were counted to construct a per-patient matrix of workload indicator counts. In cases of multiple mapping, occurrences of SNOMED CT codes induced occurrences of all associated workload indicators. Similarly, 93 long-term conditions were mapped to 12 948 SNOMED CT codes to construct a per-patient matrix of long-term condition counts. Analyses were restricted to patients with at least two recorded long-term conditions and at least one workload indicator. Comprehensive annotated lists of the clinical codes are provided in online supplemental files.

### Clustering analysis

The K-means algorithm was used to construct a typology of patients using the untransformed matrix of workload indicator counts. To determine the optimal size of the typology (*k*), we computed the mean silhouette index associated with each value of *k* within a workable range. This index compares, for each patient, the mean distance from patients within the same cluster to the mean distance from patients belonging to the closest cluster. This index varies from −1 to 1, higher values indicating that the patient is closer to other patients from the same cluster. For *k* varying in a range of workable number of clusters, the model was trained on the full dataset, and cluster assignments were obtained. At each iteration, a random subsample of 20 000 data points was drawn, the mean silhouette score being computed from this sample. This clustering procedure is intended to identify a workable number of clusters, balancing interpretability with an acceptable silhouette index.

### Statistical analysis

The corresponding author had full access to the data and accepts responsibility for submission. Patients’ follow-up was calculated from first record to death or last record. Incidence of long-term conditions was estimated as first recorded occurrences per patient-year. Frequency of workload indicators was estimated as counts per patient-year. Patients’ demographic characteristics and long-term conditions were summarised using counts and percentages or median and inter-quartile range as appropriate. A heatmap displays the row-wise scaled values of the matrix of long-term condition incidence across workload clusters. A second heatmap displays the row-wise scaled values of the matrix of workload indicator frequencies across workload clusters. For descriptive purposes, a secondary clustering of long-term conditions was conducted based on the row-wise scaled incidence matrix across workload clusters. All analyses were performed using R V.4.5.0.^24^

### Patient and public involvement

Patient and public involvement informed the development of the workload framework used in this study. The set of workload indicators was derived from a previously published qualitative evidence synthesis and consensus process involving patients. No patients or members of the public were directly involved in the conduct of the present analysis.

## Results

### Patients

From an initial cohort of 14 785 237 patients, randomly sampled from the CPRD, 1 258 248 patients, born between 1 January 1956 and 31 December 1965, were identified. Among them, 765 930 had at least two recorded long-term conditions and at least one record of workload indicator. Table 1 displays analysed patients’ characteristics. Briefly, the median age at first record was 31 years (IQR 23–38) and the median follow-up was 28 years (IQR 19–37). Of the 765 930 patients, 49.4% were male (n=378 154) and 50.6% were female (n=387 769). Patients were predominantly of white (68.1%, n=521 859) ethnicity, followed by patients of Asian (4.5%, n=34 249), black (3.1%, n=23 502), other (2.0%, n=15 290) or mixed ethnicity (0.4%, n=3264).

**Table 1:** Patient characteristics

Characteristic	Category	Total
Total N		765 930
Ethnicity	White	521 859 (68.1%)
	Black	23 502 (3.1%)
	Asian	34 249 (4.5%)
	Mixed	3264 (0.4%)
	Others	15 290 (2.0%)
	Missing	167 766 (21.9%)
Sex	Male	378 154 (49.4%)
	Female	387 769 (50.6%)
	Missing	7 (0.0%)
Age at first record	Median (IQR)	31.0 (23.0–38.0)
Follow-up, years	Median (IQR)	28.0 (19.0–37.0)
No. distinct workload indicators	Median (IQR)	15.0 (11.0–19.0)
No. distinct workload themes	Median (IQR)	5.0 (4.0–5.0)
No. distinct long-term conditions	Median (IQR)	4.0 (2.0–5.0)

### Workload domains, workload indicators and long-term conditions

Among the 10 263 SNOMED CT codes mapped to the 63 workload indicators, 306 (3.0%) mapped to multiple indicators (299 to two workload indicators and 7 to three workload indicators), and 378 (3.7%) also mapped to a long-term condition. The median number of distinct workload indicators and distinct long-term conditions was 15 (IQR 11–19) and 4 (IQR 2–5), respectively (table 1). The number of records associated with any workload indicator was 2.86 per patient-year, and the incidence of any distinct long-term conditions was 0.14 per patient-year (online supplemental table S1). The incidence of all 93 long-term conditions across clusters is provided in online supplemental table S3.

### Clustering analysis

The silhouette index across a range of typology sizes is shown in online supplemental figure S1. This graph indicates that, after an initial decrease in the silhouette index for *k*=2–4, the mean silhouette index plateaus, from *k*=4 to *k*=9, and decreases thereafter. Choosing *k*=9 represents therefore an acceptable compromise before the silhouette index deteriorates. The resulting clusters were labelled from 1 to 9 in descending order of size, ranging from 38.6% (n=295 398) to 0.2% (n=1895) of the analysed population (shown in online supplemental figure S2). To assess the consistency of the nine-cluster typology, alternative clustering solutions were subsequently obtained for *k*=7, 8, 10 and 11, and patient cluster membership was cross-tabulated against the nine-cluster solution (online supplemental table S4–S7).

### Cluster characterisation

Figure 1 and online supplemental tables S1–S3 were used to characterise the nine patient workload clusters, spanning a gradient from low to high workload intensity.

**Figure 1:**
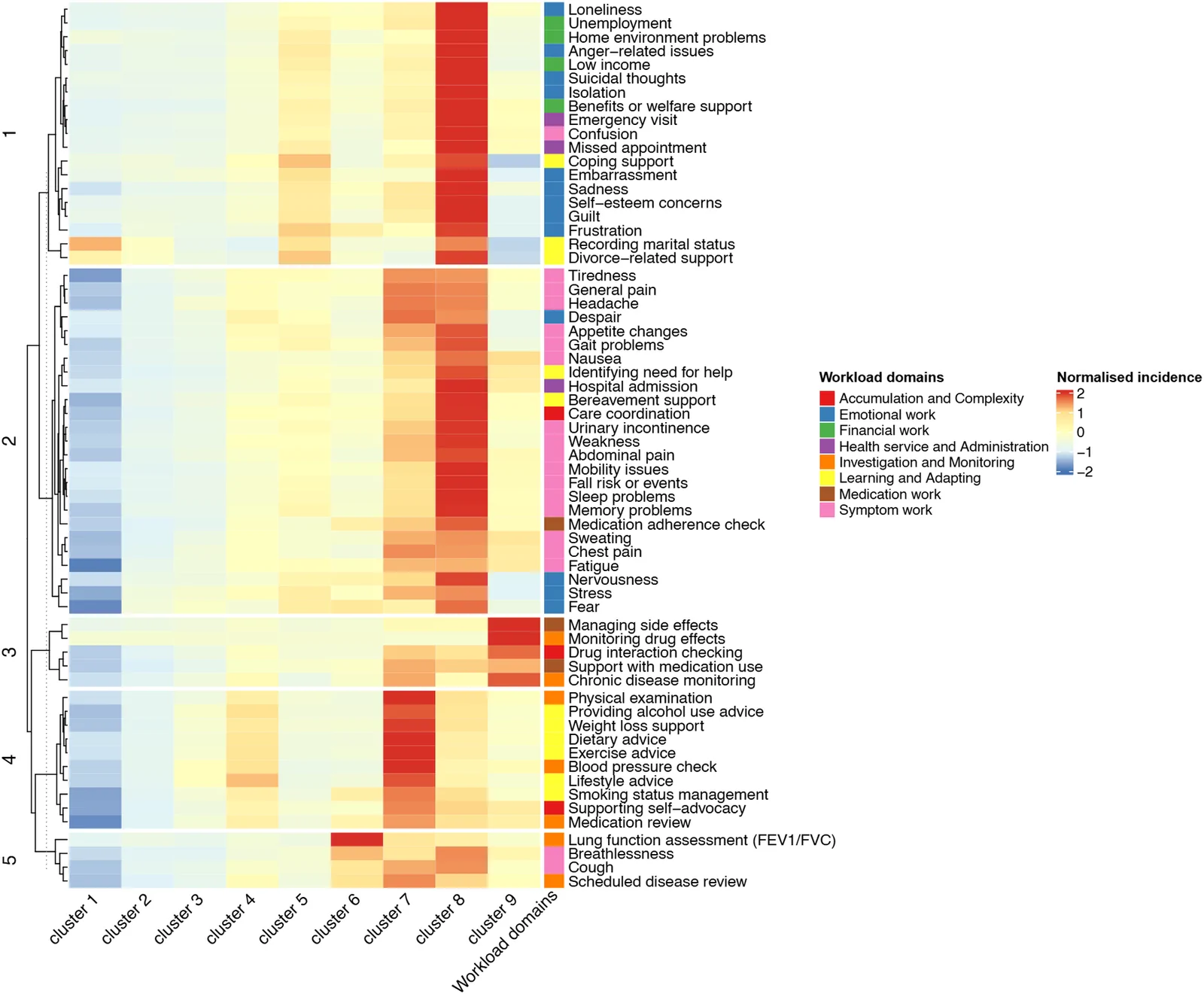
Normalised counts of workload indicators per patient-year across clusters. FEV1, forced expiratory volume in 1 second; FVC, forced vital capacity.

Cluster 1 (38.6%, n=295 398) represents a low-workload group with minimal recorded workload indicators and low incidence of long-term conditions overall, despite overrepresentation of selected conditions such as depression, anxiety, HIV and metastatic cancer.

Clusters 2 and 3 (41.5% combined, n=317 578) represent low-to-moderate workload intensity. Cluster 2 has no dominant clinical focus, whereas cluster 3 shares similar demographic characteristics but exhibits a moderate workload focus on hypertension management and preventive care.

Clusters 4–6 (16.8% combined, n=128 372) represent intermediate workload profiles with higher-than-average workload frequency and morbidity burden. These clusters differ by dominant clinical pattern: cardiometabolic and hypertension-related care (cluster 4), heterogeneous systemic and neuropsychiatric conditions with learning and adaptation work (cluster 5) and respiratory disease with monitoring-focused workload (cluster 6).

Clusters 7 and 8 (3.0% combined, n=22 687) capture among the highest workload intensity and morbidity burden, with extensive multimorbidity, high frequencies of workload indicators across multiple domains and strong overrepresentation of psychosocial, administrative and medication-related work.

Cluster 9 (0.2%, n=1895) represents a rare but distinct profile characterised by high-intensity monitoring and treatment management workload, driven by congenital/genetic cardiovascular and systemic inflammatory conditions, despite intermediate multimorbidity counts.

### Long-term conditions and clusters of workload

Figure 2 displays the heatmap of scaled incidence rate of long-term conditions across clusters of workload. Accordingly, analysed long-term conditions can be classified in five clusters.

**Figure 2:**
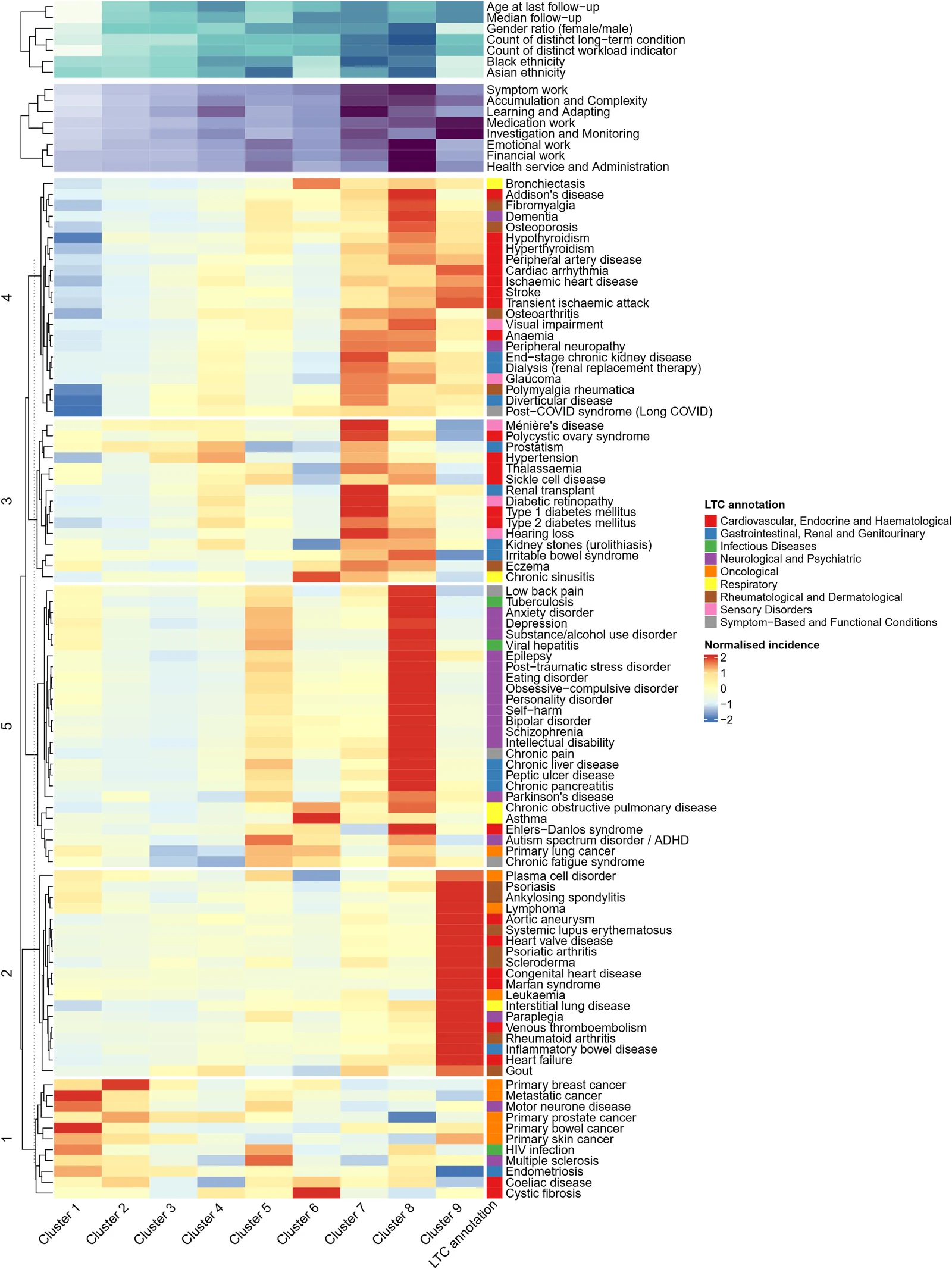
Normalised incidence of the 93 long-term conditions across clusters. Elements of online supplemental tables S1 and S2 are represented in the two upper heatmaps. ADHD, attention deficit hyperactivity disorder; LTC, long-term condition.

Condition cluster 1 comprises predominantly oncological, neurological and systemic or immune-mediated conditions. Although clinically heterogeneous, this cluster is primarily associated with workload clusters 1 and 2, which are characterised by low overall workload intensity.

Condition cluster 2 is clinically heterogeneous and dominated by chronic respiratory and airway disease, with additional endocrine/metabolic and multisystem conditions, inflammatory/atopic disease and symptom-based syndromes. It characterises workload clusters 6–8 and, to a lesser extent, clusters 5 and 9.

Condition cluster 3 encompasses mental and behavioural disorders alongside neurological, respiratory, infectious and systemic conditions. This cluster is mainly associated with workload cluster 8, characterised by high levels of health service use, symptom burden, emotional work, accumulation and complexity, and financial workload, and to a lesser extent workload cluster 5.

Condition cluster 4 is characterised by cardiovascular and cerebrovascular disease, musculoskeletal and age-related conditions, endocrine and renal disorders, neurological and cognitive impairment, and chronic systemic conditions. It is mainly associated with workload clusters 7 and 8 and, to a lesser extent, clusters 4, 5 and 9, all of which involve high multi-theme workload.

Condition cluster 5 is dominated by autoimmune, inflammatory, haematological and congenital or structural cardiovascular diseases. This cluster is strongly specific to workload cluster 9, which is characterised by intensive medication management and investigation-related workload.

## Discussion

In this population-based study of over 760 000 adults with multiple long-term conditions in a large UK primary care database, we have identified nine workload clusters. These clusters differed markedly in size, demographic composition, patterns of long-term conditions, as well as intensity and composition of workload indicators and themes. For instance, the frequency of workload indicators across the eight workload themes ranged from 1.15 records per patient-year in cluster 1 to up to 8 records per patient-year in clusters 7–9. Overall, the findings provide novel empirical evidence that recorded indicators of patient workload are concentrated within a selective group of adults living with multiple long-term conditions, challenge prevailing conceptual models of patient complexity and position patient workload as a distinct dimension of the patient experience.

### Key findings

A notable finding of our study was the consistent inverse relationship between cluster size and workload intensity. The largest clusters, incorporating the vast majority of patients, were characterised by low frequencies of workload indicators, limited engagement across workload domains and low counts of long-term conditions. By contrast, a minority of patients experienced substantially higher, multidimensional workload, with the smallest clusters accounting for the highest rates of workload indicators and the broadest engagement across workload themes. This pattern extended beyond simple disease counts, highlighting the importance of considering patient work as a distinct and clinically relevant construct beyond condition-based descriptions of patients’ health status.

The identified workload clusters are informative with regard to ongoing debates around the conceptualisation of patient complexity.^25^ Under the quantitative perspective,^15,26^ clusters broadly align along a gradient of increasing complexity, with generally higher rates of long-term conditions and greater workload intensity in smaller clusters. Yet, our findings revealed that workload and disease burden do not necessarily coincide, challenging the assumption that a higher number of long-term conditions necessarily translates into greater patient complexity.^6,9,26^ This divergence is evident in the comparison between clusters 8 and 9. Cluster 8 combined high long-term condition rate (eg, neuropsychiatric conditions) with extensive emotional, financial and administrative recorded workload indicators. This pattern is compatible with conceptual definitions of complexity that emphasise an imbalance between patient workload and capacity, in which mental health conditions act as both drivers and amplifiers of care burden.^6,9^ In this specific cluster, workload appears cumulative, multidimensional and potentially destabilising, consistent with previous descriptions of complex patients in primary care.^27,28^

Cluster 9, on the other hand, presented with the highest overall workload frequency despite only an intermediate rate of long-term conditions. Its workload profile was dominated by a limited number of clinical activities related to medications, investigations and monitoring, reflecting care for rather less common, congenital or systemic conditions with well-established clinical pathways.^5^ Notably, this cluster had low prevalence of mental health conditions and minimal emotional, health service or financial workload and does not show apparent signs of complexity.^27^ In this cluster, high care intensity and associated work did not coincide with traditional markers of reduced patient capacity, illustrating that clinically demanding care does not univocally equate to patient complexity. While these findings reinforce pragmatic frameworks in which complexity arises not from workload alone, but from the interaction between workload and contextual factors,^29^ they also highlight limitations of theoretical models of complexity that assume an erodible patient capacity in clinically burdensome disease profiles.

Other clusters occupied intermediate positions along this spectrum. Cluster 7, for example, combined high workload with cardiometabolic and renal disease but displayed a more balanced distribution across workload domains, suggesting relative stability and adaptation.^27^ Larger clusters showed close alignment between long-term condition profiles and expected workload, with little evidence of increased burden. Together, these patterns suggest that workload-based typologies capture dimensions of patient experience that are not fully represented by disease-based classifications alone.^13,27,28,30,31^

Our workload-oriented approach also produced a contrasted typology of long-term conditions. Beyond its practical usefulness for characterising patient workload clusters and revealing their underlying clinical profiles, this typology generally aligns with established multimorbidity patterns that include cardiometabolic, mental health and respiratory clusters.^4,32,33^ Of particular interest, however, is long-term condition cluster 5, which specifically characterises patient workload cluster 9. This association illustrates the link between the clinical profile of this cluster—characterised by congenital or systemic conditions—and a restricted number of technical workload indicators that define the most burdensome patient cluster in our study. Of note, this cluster of long-term conditions excludes cardiometabolic risk factors (condition cluster 4) and neuropsychiatric disorders (condition cluster 3) that characterise burdensome patient clusters 7 and 8.

In contrast, patient workload cluster 8, characterised by long-term condition cluster 3 with higher incidence of neurological and psychiatric long-term conditions, also presented higher counts of emotional work. This pattern may partly reflect more frequent healthcare contact among patients with mental health conditions, providing greater opportunities for workload indicators to be assessed and recorded, as previously suggested,^13^ but also residual overlap from codes mapping to both workload and mental health conditions (online supplemental table S8).

### Implications

Multimorbidity and the burden of treatment have long been acknowledged as central features of contemporary care, prompting growing interest in whether treatment burden should be regarded not only as a patient experience but also as an indicator of the quality of care.^34^ Accordingly, current guidance on multimorbidity emphasises individualised management of multimorbidity, with explicit consideration of treatment burden and patient capacity, and a focus on rationalising care to minimise demands and fragmentation of care.^5^ Our findings provide new evidence supporting these recommendations, by showing that workload and care demands vary substantially across people living with multimorbidity and cannot be inferred from disease profiles alone. Although our results also show that treatment burden cannot be simply assimilated to patient complexity, the identification of groups of patients whose care involves substantial and sustained work invites closer attention to how such workload is experienced and managed, even when care is well organised and does not exhibit features traditionally associated with complexity.

Conversely, burdensome clusters aligning with widespread conceptualisations of patient complexity in which mental health conditions play a central role lend support to more integrated models of care, placing less focus on optimisation of disease-specific management and greater emphasis on prioritisation, continuity and integration of mental health within care pathways, in line with current recommendations.^3,5,27^

These findings also have implications for the conceptualisation, measurement and management of patient complexity and patient workload within health systems. Although treatment burden is recognised in policy and guidance,^5^ it is not directly observable in current routine indicators, which primarily describe conditions and delivered care rather than patient work. The workload-based approach makes visible this dimension of care demand, allowing identification of patient groups in which care demands accumulate. The observed concentration of high workload among a minority of patients indicates scope for policy action with clear potential benefit for both patients and the health system. Targeted investment in care coordination, mental health integration and social support for these groups may improve patient experience and better align services with patient need. Furthermore, the association between high workload and demographic factors—such as sex and ethnicity in clusters 7 and 8—emphasises the importance of equity-focused services and highlights the potential role of workload-based measures in monitoring and addressing health inequalities. These priorities are consistent with those set out in the NHS Long Term Plan.^35^

### Strengths and limitations

Our study draws on a large, nationally representative primary care dataset with long follow-up. The proposed approach is founded on the working hypothesis, developed through previous conceptual and methodological work informed by qualitative evidence synthesis, that beyond the simple count of long-term conditions, various other aspects of patients’ lived experience can be captured through routinely collected electronic health records. This framework explicitly assumes, for instance, that prolonged and frequent contact with healthcare services reflects workload associated with navigating and engaging with healthcare, as captured by the cumulative count of recorded indicators within the Health Service and Administration domain. This concept of workload accumulation through prolonged and frequent contact with healthcare services, for instance, also informed the choice to conduct the analysis using cumulative counts of workload indicators rather than incidence measures, which are better suited to describing the occurrence of long-term conditions. This approach tends to distinguish patients with a sustained intensity of workload indicators from patients experiencing the same intensity over a shorter period, while patients with shorter follow-up also tend to be clustered together, as illustrated by cluster 1.

Of note, limitations associated with the proposed approach include potential changes and variations in clinical coding practices over the study period and possible coding errors, possible discrepancies between workload constructs and recorded workload indicators, residual inflation of workload indicator counts due to multiple mapping of codes across workload indicators and residual overlap between indicators identifying both workload and long-term conditions. The absence of a measure of socioeconomic deprivation also limits the comprehensive characterisation of patients’ workload clusters.

The use of unsupervised methods allowed workload patterns to be identified empirically, without imposing strong prior assumptions about the underlying patient structure. Conversely, clustering results are inherently sensitive to methodological choices, and alternative specifications would yield different typologies. However, given our objective of examining patient workload quantitatively, clustering patients on counts of workload indicators using a well-established method was a coherent and appropriate analytic choice. Of note, while the silhouette values were moderate, this index reflects geometric separation in a multivariate space defined by counts of workload indicators. Given the overlapping and cumulative nature of such count data across patients, strong geometric separation was not necessarily expected. In this context, the silhouette index was used to inform the selection of a workable level of granularity in the clustering solution, rather than to demonstrate strong geometric separation between groups, and does not by itself determine the substantive or clinical coherence of the identified groups. Nevertheless, the sensitivity analysis conducted using alternative values of *k* showed that small clusters were generally well preserved across the tested range, in particular cluster 9, which was preserved in the 7-cluster, 8-cluster and 10-cluster solutions and was cleanly distributed across two subclusters in the 11-cluster solution.

Yet, although a gradient in workload intensity across the identified clusters could be anticipated, the contrasts in long-term condition profiles and demographic structure were not, revealing clinical and demographic heterogeneity beyond the gradient of workload counts. While this approach emphasises the quantitative and cumulative aspects of workload, the resulting imbalanced typology was consistent with previously described long-term condition clusters,^4,32,33,36^ lending empirical relevance to smaller patient groups. Finally, we acknowledge that alternative clustering approaches that more explicitly account for the longitudinal structure of electronic health records^37,38^ may identify complementary workload patterns and warrant further investigation.

## Conclusion

Our study illustrates the value of constructed workload indicators and a patient-oriented approach for characterising real-life workload profiles among patients with multiple long-term conditions. By characterising clusters of patients with distinct profiles in terms of long-term conditions, demographic characteristics and markers associated with patient complexity, our study suggests that patient workload, as reflected by recorded indicators of patient workload, constitutes a distinct dimension of the patient experience that does not reduce to a simple component of patient complexity. In particular, the identification of patients experiencing high workload in the absence of features traditionally associated with complexity points to policy action with potential benefit for both patients and the health system. Across other high-workload clusters characterised by traditional signs of complexity, the prominence of mental health conditions and their associated workload reinforces the central role of mental health within policies addressing patient complexity and multimorbidity.

## Supplementary material

10.1136/bmjph-2026-005763 online supplemental file 1

## Data Availability

Data may be obtained from a third party and are not publicly available.
