# Supplementary material for: Patient-centred workload clustering in adults under 65 with long-term conditions from longitudinal primary care data
Source: BMJ Public Health. 2026 Oct 30;4(4):e005763. doi: 10.1136/bmjph-2026-005763 (PMC13723894; doi:10.1136/bmjph-2026-005763)
Supplement: Supplementary file 1 [file bmjph-4-4-s001.pdf]

Patient-Centred Workload Clustering in Midlife with Long-Term Conditions using Primary Care Data at Scale

Supplementary Material

Marc Delord[1,*], Emilia Holland[2], Mozhdeh Shiranirad[3], Alexandru Dregan[4], and Simon D. S. Fraser[2,5,6]

[1]School of Life Course & Population Sciences„ Department of Population Health Sciences, King's College London, London, UK
[2]School of Primary Care, Population Sciences and Medical Education, Faculty of Medicine, University of Southampton, Southampton, UK
[3]School of Mathematical Sciences, University of Southampton, Southampton, UK
[4]Department of Psychological Medicine, Institute of Psychiatry, Psychology and Neuroscience, King's College London, London, UK
[5]NIHR Applied Research Collaboration Wessex, Southampton, UK
[6]University Hospital Southampton NHS Foundation Trust, Southampton, UK
[*]Correspondence: marc.delord@kcl.ac.uk, Tel: +44 20 7848 8710

Additional Clustering procedure information

The number of clusters was chosen after computing the silhouette index for clustering solutions ranging from (k=2) to (k=20), using k-means clustering on the untransformed matrix of workload indicator counts with squared Euclidean distance. Because calculation of the silhouette coefficient on the full dataset was computationally prohibitive, it was calculated using a random subsample of 20,000 patients drawn without replacement. A new random subsample of 20,000 patients was drawn for each value of (k). After examination of the silhouette scores, the final typology was computed on the full untransformed matrix of workload indicator counts using squared Euclidean distance with the native R kmeans function, with a random seed of 1 and 50 random starts.

The secondary clustering of long-term conditions displayed in Figure 2 was derived from normalised incidence calculated within each patient cluster, as displayed in Supplementary Table S3, and standardised across clusters for each long-term condition. Following visual inspection of the k-means clustering of long-term conditions, a five-cluster solution was considered convenient for describing patient clusters.

Table S1: *Patients characteristics*

Cluster #		1	2	3	4	5	6	7	8	9	Total
Total N (%)		295398 (38.6)	217454 (28.4)	100124 (13.1)	49269 (6.4)	40870 (5.3)	38233 (5.0)	13049 (1.7)	9638 (1.3)	1895 (0.2)	765930
Ethnicity	White	179771 (60.9)	162242 (74.6)	73561 (73.5)	33980 (69.0)	26261 (64.3)	30114 (78.8)	8385 (64.3)	6007 (62.3)	1538 (81.2)	521859 (68.1)
	Black	7749 (2.6)	5531 (2.5)	3326 (3.3)	2317 (4.7)	2723 (6.7)	531 (1.4)	654 (5.0)	658 (6.8)	13 (0.7)	23502 (3.1)
	Asian	9935 (3.4)	8651 (4.0)	5111 (5.1)	3931 (8.0)	3128 (7.7)	1253 (3.3)	1355 (10.4)	858 (8.9)	27 (1.4)	34249 (4.5)
	Mixed	1496 (0.5)	799 (0.4)	351 (0.4)	172 (0.3)	269 (0.7)	82 (0.2)	39 (0.3)	51 (0.5)	5 (0.3)	3264 (0.4)
	Others	7825 (2.6)	3780 (1.7)	1563 (1.6)	545 (1.1)	816 (2.0)	469 (1.2)	134 (1.0)	134 (1.4)	24 (1.3)	15290 (2.0)
	(Missing)	88622 (30.0)	36451 (16.8)	16212 (16.2)	8324 (16.9)	7673 (18.8)	5784 (15.1)	2482 (19.0)	1930 (20.0)	288 (15.2)	167766 (21.9)
Sex	Male	163755 (55.4)	100623 (46.3)	46103 (46.0)	23707 (48.1)	17618 (43.1)	16203 (42.4)	5488 (42.1)	3621 (37.6)	1036 (54.7)	378154 (49.4)
	Female	131639 (44.6)	116830 (53.7)	54021 (54.0)	25562 (51.9)	23252 (56.9)	22028 (57.6)	7561 (57.9)	6017 (62.4)	859 (45.3)	387769 (50.6)
	(Missing)	4 (0.0)	1 (0.0)	0 (0.0)	0 (0.0)	0 (0.0)	2 (0.0)	0 (0.0)	0 (0.0)	0 (0.0)	7 (0.0)
Age at registration	Median (IQR)	32.0 (24.0 to 40.0)	31.0 (23.0 to 38.0)	30.0 (23.0 to 37.0)	29.0 (21.0 to 36.0)	31.0 (23.0 to 39.0)	27.0 (16.0 to 34.0)	27.0 (18.0 to 34.0)	29.0 (20.0 to 36.0)	28.0 (18.0 to 35.0)	31.0 (23.0 to 38.0)
Follow-up, years	Median (IQR)	22.0 (14.0 to 32.0)	30.0 (22.0 to 37.0)	31.0 (24.0 to 39.0)	33.0 (26.0 to 41.0)	29.0 (21.0 to 38.0)	35.0 (28.0 to 46.0)	35.0 (29.0 to 45.0)	33.0 (26.0 to 42.0)	35.0 (27.0 to 44.0)	28.0 (19.0 to 37.0)
No. distinct workload indicator	Median (IQR)	10.0 (7.0 to 13.0)	15.0 (13.0 to 18.0)	17.0 (15.0 to 20.0)	20.0 (17.0 to 23.0)	19.0 (16.0 to 23.0)	20.0 (18.0 to 24.0)	24.0 (21.0 to 28.0)	25.0 (21.0 to 29.0)	20.0 (16.0 to 24.0)	15.0 (11.0 to 19.0)
No. distinct workload theme	Median (IQR)	4.0 (3.0 to 5.0)	5.0 (4.0 to 5.0)	5.0 (4.0 to 6.0)	5.0 (5.0 to 6.0)	5.0 (5.0 to 6.0)	5.0 (5.0 to 6.0)	6.0 (5.0 to 7.0)	6.0 (6.0 to 7.0)	5.0 (4.0 to 6.0)	5.0 (4.0 to 5.0)
No. distinct long-term condition	Median (IQR)	3.0 (2.0 to 4.0)	4.0 (3.0 to 5.0)	4.0 (3.0 to 6.0)	6.0 (4.0 to 8.0)	6.0 (4.0 to 7.0)	6.0 (4.0 to 8.0)	8.0 (6.0 to 11.0)	9.0 (7.0 to 11.0)	6.0 (4.0 to 9.0)	4.0 (2.0 to 5.0)

Table S2: *Counts of records of workload indicators per domains across clusters (per patient-year)*

Cluster #	1	2	3	4	5	6	7	8	9	All
Investigation and Monitoring	0.482	1.148	1.940	3.234	1.618	2.564	5.192	3.415	7.016	1.482
Learning and Adapting	0.326	0.573	0.857	1.392	0.766	0.988	1.956	1.415	0.923	0.685
Health service and Administration	0.169	0.254	0.285	0.465	1.053	0.403	0.777	2.302	0.794	0.349
Symptom work	0.124	0.222	0.261	0.365	0.370	0.431	0.657	0.744	0.424	0.249
Emotional work	0.040	0.056	0.059	0.076	0.110	0.082	0.120	0.177	0.065	0.061
Accumulation and Complexity	0.008	0.015	0.020	0.031	0.025	0.026	0.047	0.046	0.036	0.018
Financial work	0.004	0.005	0.005	0.011	0.021	0.015	0.023	0.050	0.012	0.008
Medication work	0.001	0.004	0.005	0.011	0.008	0.013	0.020	0.023	0.030	0.005
Total	1.155	2.277	3.433	5.585	3.971	4.522	8.791	8.170	9.300	2.857

Table S3: *Incidence of long-term conditions (per 10,000 patient-year)*

Cluster #	1	2	3	4	5	6	7	8	9	All
Low Back Pain	156.00	145.09	142.77	147.39	167.63	135.07	161.92	187.75	130.69	150.01
Depression	147.18	115.87	101.02	108.88	174.21	125.44	131.84	197.10	111.66	128.54
Hypertension	52.62	98.45	197.81	222.61	101.29	84.65	216.61	151.95	124.30	111.34
Anxiety	109.17	93.91	85.21	87.68	134.42	96.27	100.13	153.00	80.97	100.44
Osteoarthritis	70.77	81.47	87.67	100.73	98.86	86.27	118.16	120.34	92.76	83.33
Eczema	77.47	68.66	66.92	70.79	68.14	81.13	88.25	85.76	68.47	72.83
Asthma	55.51	54.73	34.66	51.66	59.06	189.44	95.39	108.86	58.10	62.58
Substance Use Disorder	66.85	47.24	43.74	47.47	91.15	49.82	49.72	108.86	44.32	56.58
Type 2 Diabetes Mellitus	22.76	35.54	63.56	132.41	85.40	48.47	170.49	143.76	65.35	51.74
Hearing Loss	39.20	38.37	38.21	41.61	40.43	39.15	53.01	50.19	41.05	39.55
Peripheral Neuropathy	31.11	33.50	37.92	50.77	46.12	38.26	75.04	74.52	47.59	37.39
Irritable Bowel Syndrome	35.15	34.34	33.84	33.37	35.35	35.57	40.97	43.67	27.42	34.86
Hypothyroidism	20.78	29.27	28.16	29.10	32.52	29.66	34.74	39.59	35.23	26.96
Diverticular Disease	18.39	23.90	27.62	29.66	25.36	27.65	34.45	30.30	29.69	23.86
Anaemia	17.31	19.55	21.98	31.01	30.93	21.18	52.31	51.45	37.50	22.14
Psoriasis	24.02	19.65	19.39	20.39	21.09	18.08	22.21	24.91	31.82	21.20
Diabetic Retinopathy	5.66	10.98	24.41	72.99	33.20	16.67	115.61	72.25	30.12	21.13
Venous Thromboembolism	18.99	18.08	18.44	22.18	29.07	20.12	30.72	41.86	107.39	20.43
Ischaemic Heart Disease	9.47	16.96	27.16	37.92	26.62	20.19	50.92	47.83	56.11	19.88
Gout	17.67	18.26	23.77	28.43	16.53	14.10	30.22	19.75	34.38	19.65
Chronic Obstructive Pulmonary Disease	9.00	14.97	10.02	18.62	35.88	65.75	37.36	73.59	30.97	18.62
Chronic Pain	13.10	14.35	14.80	20.58	31.26	21.98	33.16	51.36	20.46	16.99
Arrhythmia	11.82	15.36	18.88	22.70	17.73	15.35	29.54	29.74	37.08	16.09
Stroke	10.25	12.06	16.19	20.87	22.04	12.79	28.53	34.87	39.35	14.22
Epilepsy	13.24	10.67	8.39	9.85	20.12	12.36	12.54	26.74	17.19	12.04
Osteoporosis	9.13	12.08	10.82	11.30	18.03	17.00	16.85	24.53	19.46	11.90
Chronic Sinusitis	9.32	10.68	10.62	11.27	9.81	14.86	13.62	11.77	8.95	10.60
Primary Malignant Skin Cancer	12.20	11.22	10.32	8.31	6.92	8.30	7.53	6.61	12.22	10.58
Urolithiasis	10.33	9.06	10.08	12.11	9.94	7.31	13.08	13.05	10.65	9.94
Prostatism	9.32	9.77	9.60	10.34	7.93	8.19	10.41	9.06	8.52	9.44
Primary Malignant Breast Cancer	9.28	10.57	8.42	7.05	7.94	8.53	6.71	6.99	6.96	9.14
Type 1 Diabetes Mellitus	3.30	3.67	6.93	27.04	13.21	5.59	58.62	39.74	15.34	8.35
Self-Harm	8.87	5.30	4.10	5.68	18.10	9.78	10.47	34.58	7.67	7.86

(Continued on Next Page...)

Table S3: *Incidence of long-term conditions (per 10,000 patient-year) (continued)*

Cluster #	1	2	3	4	5	6	7	8	9	All
Fibromyalgia	4.32	6.27	7.07	9.99	14.80	12.08	15.81	21.70	12.22	7.41
Heart Failure	2.98	5.48	8.28	13.31	11.88	7.74	23.93	25.20	50.15	7.09
Glaucoma	6.19	6.86	7.02	8.73	7.18	5.75	11.45	11.22	9.09	6.94
Endometriosis	7.63	6.80	6.73	5.83	6.33	5.70	6.00	6.79	3.69	6.85
Chronic Liver Disease	6.62	4.37	4.43	6.89	14.98	5.03	9.07	19.72	7.67	6.29
Peripheral Arterial Disease	3.40	4.74	5.96	11.02	11.96	7.14	19.77	24.35	21.73	6.25
Rheumatoid Arthritis	4.01	5.30	5.65	7.29	9.32	8.29	11.49	12.03	35.80	5.87
Heart Valve Disease	3.31	4.54	6.69	8.29	6.14	4.37	12.75	10.31	47.73	5.25
Personality Disorder	6.51	2.87	1.82	2.86	11.84	5.77	5.51	23.54	3.84	4.94
Visual Impairment	3.88	3.72	4.23	6.80	8.05	4.74	11.47	13.69	8.95	4.75
Schizophrenia	4.83	2.79	2.04	4.16	11.10	7.81	7.35	22.58	4.26	4.64
Peptic Ulcer Disease	4.58	3.72	3.66	4.88	7.43	4.01	6.48	9.64	4.97	4.46
Post-Traumatic Stress Disorder	4.74	3.64	2.78	3.59	9.06	4.55	5.03	12.61	4.26	4.39
Transient Ischaemic Attack	2.29	3.71	5.58	7.17	6.38	4.54	10.39	13.02	15.91	4.35
Metastatic Cancer	5.60	4.26	3.25	2.91	3.93	3.21	2.88	3.18	2.27	4.28
Viral Hepatitis	5.83	2.38	1.85	2.28	9.90	2.42	2.21	12.38	3.13	3.97
Eating Disorder	4.43	3.32	2.33	2.68	6.75	4.01	4.41	9.85	3.13	3.84
Bipolar Disorder	3.48	2.60	2.07	3.58	8.14	7.10	6.13	18.21	4.12	3.81
Hyperthyroidism	3.09	3.84	4.13	4.02	5.02	3.65	5.78	6.09	5.68	3.80
Intellectual Disability	3.23	2.99	2.74	3.60	5.66	4.45	4.93	7.72	3.55	3.44
Primary Malignant Prostate Cancer	3.13	3.45	3.20	3.26	2.91	2.72	2.69	2.18	2.70	3.18
Tuberculosis	3.67	2.34	2.13	2.95	5.01	2.73	3.75	6.64	2.13	3.05
Obsessive-Compulsive Disorder	3.22	2.49	1.91	2.25	5.26	3.36	3.52	7.46	2.27	2.93
Polycystic Ovary Syndrome	3.14	2.54	2.76	3.42	2.76	2.36	5.18	4.22	1.42	2.91
Primary Malignant Bowel Cancer	3.18	2.66	2.31	2.27	2.32	2.23	2.63	2.56	2.70	2.69
Chronic Fatigue Syndrome	2.72	2.57	2.34	2.25	3.18	3.07	2.73	3.18	2.84	2.64
Bronchiectasis	1.49	2.25	1.80	2.57	3.53	8.45	6.17	7.22	5.82	2.62
Congenital Heart Disease	2.47	2.21	2.71	2.45	2.01	2.11	3.15	2.83	15.20	2.43
Psoriatic Arthritis	1.94	1.98	2.48	3.12	2.81	2.12	4.22	3.76	13.78	2.30
Coeliac Disease	2.11	2.34	1.81	1.65	2.17	2.35	2.09	2.24	1.70	2.12
Chronic Pancreatitis	1.79	1.55	1.62	2.32	4.94	2.09	3.25	8.80	3.69	2.08
Multiple Sclerosis	2.34	2.18	1.62	1.28	2.77	1.63	1.28	2.07	1.99	2.06
Lymphoma	2.39	1.93	1.75	1.71	2.03	1.52	2.05	1.89	3.98	2.02
Ménière's Disease	1.82	2.01	2.05	2.04	1.76	1.79	2.65	1.95	1.42	1.94

(Continued on Next Page...)

7

Table S3: *Incidence of long-term conditions (per 10,000 patient-year) (continued)*

Cluster #	1	2	3	4	5	6	7	8	9	All
Polymyalgia Rheumatica	1.27	1.92	2.32	2.52	2.09	2.27	3.35	2.74	2.98	1.90
Dementia	1.35	1.53	1.31	1.73	3.05	1.91	3.08	4.54	3.13	1.66
Thalassaemia	1.78	1.37	1.52	1.90	2.13	0.81	2.92	2.68	1.14	1.62
Primary Malignant Lung Cancer	1.85	1.49	1.00	1.11	2.25	2.24	1.35	2.16	1.42	1.60
HIV	2.56	1.11	0.82	0.89	2.38	0.59	0.81	2.10	0.71	1.56
Aortic Aneurysm	1.13	1.44	1.83	2.24	1.76	1.58	2.57	1.86	5.97	1.54
End-Stage Chronic Kidney Disease	0.76	0.72	1.86	4.00	2.14	0.37	8.61	6.03	4.97	1.49
Ankylosing Spondylitis	1.66	1.32	1.15	1.38	1.40	1.20	1.32	1.40	2.27	1.41
Autism/ADHD	1.30	1.30	0.94	1.17	2.56	1.96	1.32	2.39	0.85	1.37
Sickle Cell Disease	1.40	0.84	1.13	1.67	2.20	0.47	2.17	2.48	0.57	1.23
Systemic Lupus Erythematosus	0.98	0.97	1.14	1.18	1.61	1.10	1.86	1.72	6.39	1.11
Leukaemia	1.16	1.06	1.04	0.97	1.12	1.04	1.28	0.87	1.99	1.09
Paraplegia	1.06	0.94	0.84	1.06	2.00	1.01	1.49	1.92	3.41	1.07
Interstitial Lung Disease	0.60	0.94	0.87	1.45	1.60	1.75	1.88	2.53	3.55	1.01
Parkinson’s Disease	0.88	1.03	0.89	0.82	1.31	0.88	1.26	1.43	1.14	0.96
Inflammatory Bowel Disease	0.75	0.98	0.92	1.15	1.06	1.15	1.18	1.60	2.98	0.95
Dialysis	0.44	0.45	1.02	2.39	1.43	0.25	5.20	4.46	3.27	0.90
Long COVID	0.35	0.84	1.05	1.16	1.10	1.30	1.41	1.40	1.14	0.81
Renal Transplant	0.29	0.31	1.01	2.05	0.64	0.11	4.31	1.60	1.85	0.66
Plasma Cell Disorders	0.47	0.44	0.40	0.36	0.51	0.29	0.37	0.41	0.57	0.43
Addison’s Disease	0.21	0.33	0.37	0.48	0.68	0.68	1.22	1.78	0.57	0.40
Scleroderma	0.22	0.27	0.32	0.26	0.36	0.24	0.43	0.29	0.71	0.27
Motor Neurone Disease	0.33	0.27	0.19	0.18	0.29	0.19	0.15	0.17	NA	0.26
Marfan Syndrome	0.14	0.14	0.13	0.09	0.07	0.10	0.08	0.12	1.28	0.13
Ehlers–Danlos Syndrome	0.12	0.09	0.12	0.12	0.19	0.22	0.06	0.32	0.14	0.12
Cystic Fibrosis	0.05	0.05	0.03	0.06	0.05	0.09	0.04	0.03	NA	0.05
Total	1274.71	1282.42	1406.45	1738.61	1791.55	1559.64	2229.02	2506.20	1856.52	1423.41

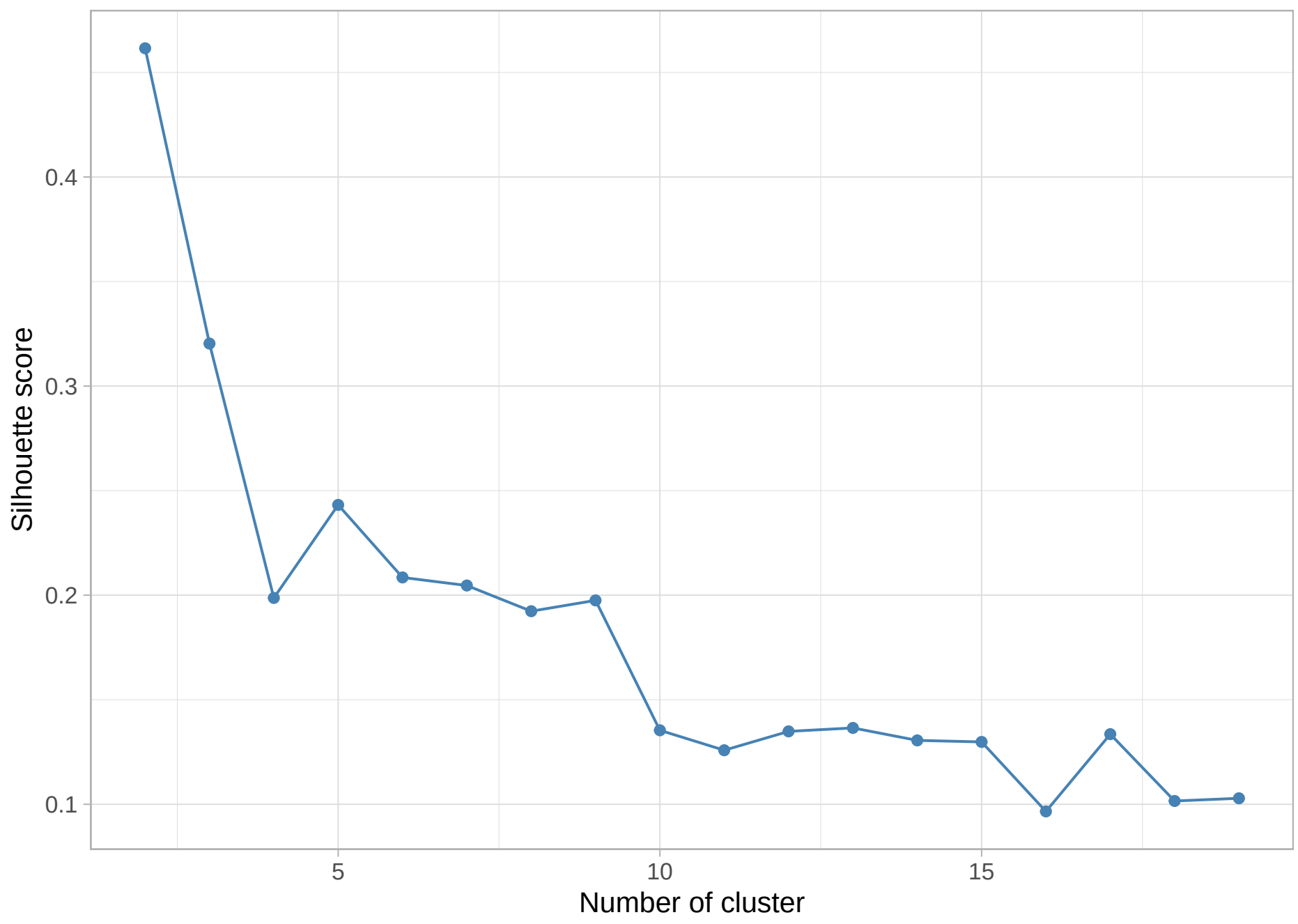

Figure S1: *Silhouette index across tested numbers of clusters*

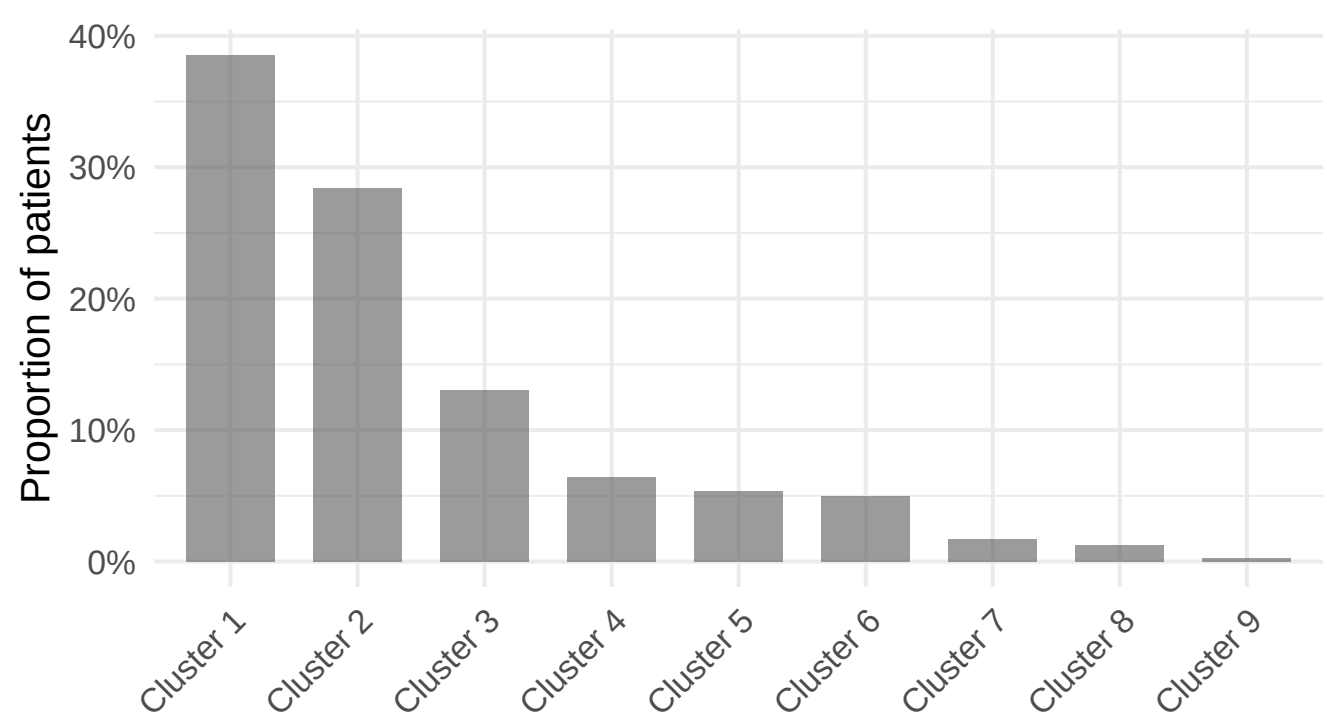

Figure S2: *Relative frequency of patients across clusters*

Table S4: *Cross-tabulation of patient cluster membership between the nine-cluster and seven-cluster solutions (%)*

	Alt.Clust. 1	Alt.Clust. 2	Alt.Clust. 3	Alt.Clust. 4	Alt.Clust. 5	Alt.Clust. 6	Alt.Clust. 7
Cluster 1	0.0	100.0	0.0	0.0	0.0	0.0	0.0
Cluster 2	0.0	24.3	0.0	0.0	75.7	0.0	0.0
Cluster 3	0.0	0.0	45.5	0.0	47.1	0.0	7.4
Cluster 4	2.4	0.0	60.2	22.9	0.0	0.0	14.6
Cluster 5	26.0	3.1	3.0	0.0	57.0	0.0	10.9
Cluster 6	0.8	0.0	0.3	0.5	5.4	0.0	92.9
Cluster 7	0.0	0.0	0.0	99.9	0.0	0.1	0.0
Cluster 8	87.6	0.0	0.0	11.8	0.0	0.1	0.5
Cluster 9	0.0	0.0	0.0	0.0	0.0	100.0	0.0

Table S5: *Cross-tabulation of patient cluster membership between the nine-cluster and eight-cluster solutions (%)*

	Alt.Clust. 1	Alt.Clust. 2	Alt.Clust. 3	Alt.Clust. 4	Alt.Clust. 5	Alt.Clust. 6	Alt.Clust. 7	Alt.Clust. 8
Cluster 1	99.3	0.7	0.0	0.0	0.0	0.0	0.0	0.0
Cluster 2	4.3	94.7	0.0	0.0	0.7	0.3	0.0	0.0
Cluster 3	0.0	0.1	0.0	0.0	98.3	0.8	0.8	0.0
Cluster 4	0.0	0.0	0.8	0.0	1.4	0.9	95.0	1.9
Cluster 5	0.4	45.7	0.0	0.0	17.6	7.6	0.0	28.7
Cluster 6	0.0	0.0	0.2	0.0	0.0	98.1	0.6	1.1
Cluster 7	0.0	0.0	93.9	0.0	0.0	0.0	6.1	0.0
Cluster 8	0.0	0.0	12.3	0.1	0.0	0.5	0.7	86.5
Cluster 9	0.0	0.0	0.0	100.0	0.0	0.0	0.0	0.0

Table S6: *Cross-tabulation of patient cluster membership between the nine-cluster and ten-cluster solutions (%)*

	Alt.Clust. 1	Alt.Clust. 2	Alt.Clust. 3	Alt.Clust. 4	Alt.Clust. 5	Alt.Clust. 6	Alt.Clust. 7	Alt.Clust. 8	Alt.Clust. 9	Alt.Clust. 10
Cluster 1	0.0	0.0	4.2	95.8	0.0	0.0	0.0	0.0	0.0	0.0
Cluster 2	0.0	0.0	93.6	0.0	5.2	1.2	0.0	0.0	0.0	0.0
Cluster 3	0.0	0.1	0.0	0.0	91.2	0.5	0.0	0.6	7.5	0.0
Cluster 4	0.0	0.2	0.0	0.0	1.4	0.1	0.0	41.6	56.5	0.0
Cluster 5	0.0	93.5	1.6	0.0	2.6	1.2	0.0	1.0	0.1	0.0
Cluster 6	0.0	0.4	0.0	0.0	1.1	87.4	0.0	10.9	0.2	0.0
Cluster 7	75.5	0.0	0.0	0.0	0.0	0.0	0.4	22.5	1.6	0.0
Cluster 8	0.2	2.7	0.0	0.0	0.0	0.0	89.0	7.9	0.2	0.0
Cluster 9	0.0	0.0	0.0	0.0	0.0	0.0	0.0	0.5	0.1	99.3

Table S7: *Cross-tabulation of patient cluster membership between the nine-cluster and eleven-cluster solutions (%)*

	Alt.Clust. 1	Alt.Clust. 2	Alt.Clust. 3	Alt.Clust. 4	Alt.Clust. 5	Alt.Clust. 6	Alt.Clust. 7	Alt.Clust. 8	Alt.Clust. 9	Alt.Clust. 10	Alt.Clust. 11
Cluster 1	0.0	0.0	0.0	95.6	0.0	0.0	4.3	0.0	0.0	0.0	0.0
Cluster 2	1.2	0.0	0.0	0.0	0.0	0.0	93.2	0.0	5.4	0.0	0.1
Cluster 3	0.5	0.6	7.7	0.0	0.0	0.1	0.0	0.0	90.8	0.0	0.2
Cluster 4	0.1	41.5	56.3	0.0	0.1	0.2	0.0	0.0	1.3	0.0	0.5
Cluster 5	1.2	0.9	0.1	0.0	0.0	93.0	1.5	0.0	2.7	0.0	0.5
Cluster 6	87.0	10.6	0.2	0.0	0.0	0.4	0.0	0.0	1.1	0.0	0.7
Cluster 7	0.0	22.2	1.3	0.0	75.2	0.0	0.0	0.0	0.0	0.4	0.8
Cluster 8	0.0	7.8	0.2	0.0	0.2	2.7	0.0	0.0	0.0	88.4	0.7
Cluster 9	0.0	0.0	0.0	0.0	0.1	0.0	0.0	28.5	0.0	0.0	71.4

Table S8: *Overlap between mental health condition codes and workload indicators*

Long-term condition	Workload indicator	# of SNOMED CT codes
Anxiety	Breathlessness	1
Anxiety	Coping	2
Anxiety	Fear	7
Anxiety	Nervousness	1
Anxiety	Stress	3
Anxiety	Sweating	1
Autism/ADHD[a]	Disease review	3
Autism/ADHD	Isolation	1
Depression	Coping	1
Depression	Disease review	2
Depression	Medication review	1
Depression	Sadness	3
PTSD[b]	Stress	5
Personality disorder	Frustration	2
Schizophrenia	Stress	1

[a] Post-traumatic stress disorder [b] Attention-deficit/hyperactivity disorder
